# A one-health approach to surveillance of tick-borne pathogens across different host groups

**DOI:** 10.1186/s12917-025-04983-7

**Published:** 2025-10-02

**Authors:** Rachele Vada, Stefania Zanet, Anna Trisciuoglio, Amir Reza Varzandi, Andrea Calcagno, Ezio Ferroglio

**Affiliations:** 1 Department of Veterinary Sciences, University of Turin, Largo Paolo Braccini 2, Grugliasco, 10095 Italy; 2 Unit of Infectious Diseases, Department of Medical Sciences, University of Turin at “Amedeo di Savoia Hospital”, ASL “Città di Torino”, Corso Svizzer 164, Torino, 10143 Italy

**Keywords:** One health, Acarological risk, Wildlife, Tick-borne diseases, Domestic animals, Humans

## Abstract

Tick-borne pathogens are increasingly impacting wildlife and human populations’ health due to expanded vector ranges and the growing popularity of outdoor activities. This study aimed to survey the prevalence of tick-borne zoonotic (TBZ) microorganisms in a rural area, targeting different host groups involved in their circulation, namely wildlife, domestic animals, humans and questing ticks, addressing the risk associated to hunting and outdoor leisure activities in an alpine environment. The study was performed in an alpine district in Northwestern Italy. We collected 124 pools of questing ticks using dragging transects across 38 locations distributed in the district. In addition, we obtained 133 spleen samples from game ungulate species (wild boar, red deer, and chamois), 67 blood samples from volunteer rural inhabitants, and samples from 38 privately-owned hunting dogs. All samples were tested by PCR for *Babesia divergens/B. capreoli*, *B. microti-*like, *B. venatorum*, *Borrelia burgdorferi* sensu latu, *Rickettsiae Spotted Fever Group* (SFG) and *Anaplasma phagocytophilum*. All investigated pathogens, except *B. burgdorferi* s.l. (found only in questing ticks), were detected in rural inhabitants. *Babesia divergens/B. capreoli* and *B. venatorum* were highly prevalent in wild ruminants, while *B. microti*-like organisms appeared sporadically in wild boar and dogs. SFG *Rickettsiae* were most common in dogs, with occasional cases in wild boar. *Anaplasma phagocytophilum* showed low prevalence in ticks and wildlife, but higher detection rates in humans and dogs. Our results underscore the interconnected nature of tick-host dynamics in the spread of TBZ and highlight the need for an expanded surveillance approach, potentially including other significant reservoirs.

**Supplementary Information:**

The online version contains supplementary material available at 10.1186/s12917-025-04983-7.

## Background

Among the tick species present in Europe and Italy, *Ixodes ricinus* is the most widespread in natural environments [1–3]. *I. ricinus* is the principal vector for the majority of tickborne pathogens in the region, with the notable exception of spotted fever group (SFG) *Rickettsiae*, which are predominantly transmitted by other genera, including *Rhipicephalus*, also known as dog tick or kennel tick, and *Dermacentor* [1, 4]. The most frequently reported zoonotic pathogens in Europe and northern Italy include *Babesia* spp., *Anaplasma phagocytophilum*, *Borrelia burgdorferi* sensu lato, and SFG *Rickettsiae* [5–7]. Beyond the influence of the immunity status, clinical cases show how these agents represent a risk for human health [7]. All four pathogens may infect domestic dogs [8–11]; while the most clinically relevant Babesia species in canids is the non-zoonotic *Babesia canis*, *B. microti*-like strains have occasionally been detected in dogs [9–12].

Different wild species may act as reservoirs for TBZ, depending on the specific pathogen. While small mammals such as rodents, insectivores, or even birds maintain the causative agents of Lyme disease or Mediterranean Spotted Fever in the environment [1], red deer (*Cervus elaphus*) is considered a potential reservoir for *Babesia diverges* [13], reaching prevalences up to 27% in Czech Republic [14] and, together with wild boar (*Sus scrofa*), for *Anaplasma phagocytophilum* [15, 16]. The same pathogen has also been detected at a high prevalence (77.8%) in alpine chamois (*Rupicapra rupicapra*) [16]. Moreover, the presence of deer species and red fox (specifically, temporal occupancy of a site) has been shown to increase questing ticks’ abundance [17, 18], which, in return, impacts the risk of tick bite and TBZ transmission.

With tick vectors increasing in abundance and spreading geographically [19, 20], tick-borne zoonoses (TBZ) have emerged as some of the most impactful vector-borne diseases across Europe [21]. For instance, Lyme disease, presenting with a relatively high and widespread prevalence already, has increased its incidence in the past 10 years [22, 23]. Human babesiosis, another important emerging disease of global significance, is also recorded in increasing countries and prevalence Europewide [24].

In addition to the geographical expansion of tick vectors and the increase in wild host populations, the popularity of outdoor activities, further amplified by restrictions during the COVID-19 pandemic [25], is increasing the risk of tick bite for humans along facilities in the natural environment (e.g [26–28]. and beyond, and ultimately the transmission of TBZ from wildlife).

Due to their multi-host and multi-pathogen nature, tick-borne zoonoses require a comprehensive, integrated surveillance system to effectively monitor and inform decision-making for prevention strategies. Integrated surveillance data targeting TBZ encompass human and animal health, pathogen presence, vector surveillance, and environmental factors like climate and wildlife abundance [29]. Such data can also be integrated with modelling strategies, which can help predict outbreaks [30, 31]. Additionally, standardized, cross-border data collection enables data integration and minimizes bias.

In this study, we estimated the prevalence of the most common tick-borne zoonoses in Northwestern Italy, including several of the most widespread pathogens in Europe: *B. divergens/B. capreoli*, *B. microti* and *microti*-like, *B. venatorum*, *A. phagocytophilum*, *Borrelia burgdorferi sensu latu* and *Rickettsiae* causing the Mediterranean Spotted Fever (SFG *Rickettsiae*). Within an alpine setting, our analysis targeted key host groups related to the transmission through outdoor and, particularly, hunting-related activities, namely environmental ticks, rural inhabitants, hunting dogs and game ungulates. By highlighting similarities or differences in the presence of TBZ microorganisms in the different hosts, this study aimed to provide useful information to refine a comprehensive surveillance of these pathogens.

## Materials and methods

### Study area

This study was performed in an alpine hunting district in Northwestern Italy (C.A. CN3, Piedmont region, Supplementary Fig. 1). It covers an alpine valley (Valle Maira) of 567 km^2^, with altitude ranging from 500 m above the sea level up to more than 3000 m above the sea level (a.s.l.) and an alpine and continental climate. The annual precipitation in 2020 was 788.2 mm, and the mean temperature was 10.19 °C. The valley registers a population of 11.500 inhabitants (20.28 inh/km^2^) and several agropastoral activities. The hunting plan targeting ungulate species involves wild boar (*Sus scrofa*), roe deer (*Capreolus capreolus*), red deer (*Cervus elaphus*) and chamois (*Rupicapra rupicapra*).

### Sampling

The project received approval from the Ethics Committee of the University of Turin (Prot. n. 0346675, 26/06/2023 and Prot.0001785, 01/06/2023), and all participants and dog owners signed a written informed consent. All data were anonymized with unique alphanumeric code applied on samples, and Good Clinical Practice recommendations were followed. The cross-sectional study targeted the different host groups:

- Questing (environmental) ticks. Ticks were collected during August-October 2021 through 38 sampling points covering the whole valley, up to 2500 m a.s.l. At each sampling point, ticks were collected by dragging transects (a 10 m side square and a 26 m radius circle around the GPS point) and stored in 100% ethanol. They were identified with dichotomous keys [32–34] and pooled by sampling point, species, and developmental stage (pool size from 1 specimen to 56).
- Game ungulates. With the collaboration of local hunters and with the support of the hunting district management, an opportunistic collection of spleen from regularly hunted species was performed, namely wild boar, red deer, and chamois. All animals were hunted during the hunting season 2021/2022; samples were individually identified and stored at −20 °C until further analysis. The sample size for each species was proportional to the number of culled specimens available for sampling.
- Rural inhabitants. Together with game management authorities, hunters were voluntarily enrolled in the study. A sampling day was organized locally in the summer of 2023. Besides hunters, to increase the sample size, their extended families and other people related to the hunting district were also included in the cohort. All participants engaged in outdoor recreational activities at least once per week, either hunting, fishing, mushroom picking, or camping/hiking/cycling. Whole blood was collected and stored at −20 °C until further analysis.
- Dogs: participants could also voluntarily enrol their dog(s) in the study. An aliquot part of whole blood collected by routine clinical practices by private veterinarians was dedicated to this study, and tubes were stored at −20 °C until further analysis. We additionally investigated how often the animal was involved in outdoor activities (from less than once to 7 times per week) and assessed the use of antiparasitic treatment registered for Ixodidae ticks (between “never,” “sometimes,” and “always”).

### Biomolecular detection of microorganisms

Genomic DNA of vertebrate hosts was extracted from 200 ul of blood (humans and dogs) and from 10 mg of spleen (wildlife) with the PureLink Genomic DNA Mini Kit (Invitrogen, CA, USA), according to the manufacturer’s instructions. The genomic DNA of ticks was obtained by extraction from the whole pooled ticks with the blackPREP Tick DNA/RNA kit (Biosense, Italy). The concentration and quality of extracted DNA were evaluated with Nanodrop One (ThermoFisher, MA, USA). Extracted DNA was then stored at −20 °C until further analysis.

PCR protocols were implemented to detect microorganisms’ genomic DNA through all groups, despite some pathogen-hosts combinations not having been yet reported in the literature. A non-template negative control and a positive control (either DNA extracted from a cultivated pathogen or positive samples previously confirmed by amplicon sequencing) were included in all PCR reactions, and all standard precautions were taken to minimize the risk of contamination. Amplicons were analysed by agarose gel electrophoresis (2%) and visualized by staining with Gel Red Nucleic Acid Gel Stain (VWR International Milano, Italy). Primers implemented are reported in Table 1, while the complete protocols are presented in Supplementary Table 1.

**Table 1:** Primers implemented in the current work, with reference to the targeted gene and the work where the primer was firstly described

Pathogen	Gene	Reference:
*B. divergens/B. capreoli*	*18s*	[35]
*B. microti*	*18s*	[36]
*B. venatorum*	*18s*	[37]
Anaplasmataceae	* 16 s rRNA*	[38, 39]
A. phagocytophilum*	*groEL*	[40]
*B. burgdorferi* s.l.	*spacer region between 5 S and 23 S rRNA genes*	[41]
SFG Rickettsia	*surface protein rOmpA*	[42]

*To avoid false positive results due to the detection of symbiotic bacteria like Candidatus Midichloria, in ticks we implemented specific primers for A. phagocytophilum, while Anaplasmataceae primers were implemented in vertebrate hosts

Anaplasmataceae and SFG *Rickettsiae* species were further identified by sequencing (Macrogen Europe, The Netherlands) and further comparison of obtained sequences with those deposited in GenBank^®^. The targeted section of the 18 s gene for *B. divergens/B. capreoli* did not include the single-nucleotide-polymorphisms (SNPs) that allows the differentiation of the two species. However, to the best of our knowledge no zoonotic case of *B. capreoli* has been reported so far. All protocols implemented an end-point PCR, with the only exception of the one to detect *B. venatorum*, which implemented a real-time PCR.

### Statistical analysis

Prevalence with 95% confidence intervals (95% CI) in vertebrate hosts (rural inhabitants, dogs and game ungulates) was calculated through the Wilson Score method. For tick pools, prevalence was calculated as Minimum Infection Rate (MIR). Due to their zoonotic relevance, we also tested prevalence in nymphs and adults separately. To point out any statistical difference among groups, we implemented a paired comparison through a Chi-square test for vertebrate host groups [43]. Fisher’s exact test and correlation were implemented [43] to test the association between survey responses and positivity to PCR in dogs.

## Results

### Sampling population description

We sampled 532 ticks divided into 124 pools (Table 2).

**Table 2:** Number of ticks collected by species and developmental stage and corresponding number of pools

	Count	Number of pools
*Ixodes ricinus*		
Larvae	339	51
Nymphs	126	42
Adults	19	15
*Haemaphysalis punctata*		
Larvae	46	14
*Dermacentor marginatus*		
Nymphs	1	1
Adults	1	1

We sampled a total of 67 rural inhabitants, of which 54% practised hunting activities. The sex ratio of rural inhabitants was skewed towards males (49 − 18), and the age average was about 50 years.

We sampled a total of 38 dogs, with an even sex ratio between males and females and an average age of 5 years. 66.7% were engaged in outdoor activities at least once a week (33.3% less than once per week, 20.8% once per week, 8.3% twice, 29.3% three times and 8.3% daily), and seasonality in outdoor activity was linked to hunting activity (i.e. only from September to December). Antiparasitic treatment registered for Ixodidae ticks was regularly administrated in 84.4% of sampled dogs, while it was administrated at sub-optimal frequencies in 15.6% of cases.

We sampled a total of 133 game ungulates, including 117 wild boar, 13 red deer and 3 chamois. No roe deer was provided through the opportunistic sampling.

### Biomolecular detection of microorganisms – descriptive analysis and in-group comparison

#### Questing ticks

**Fig. 1:**
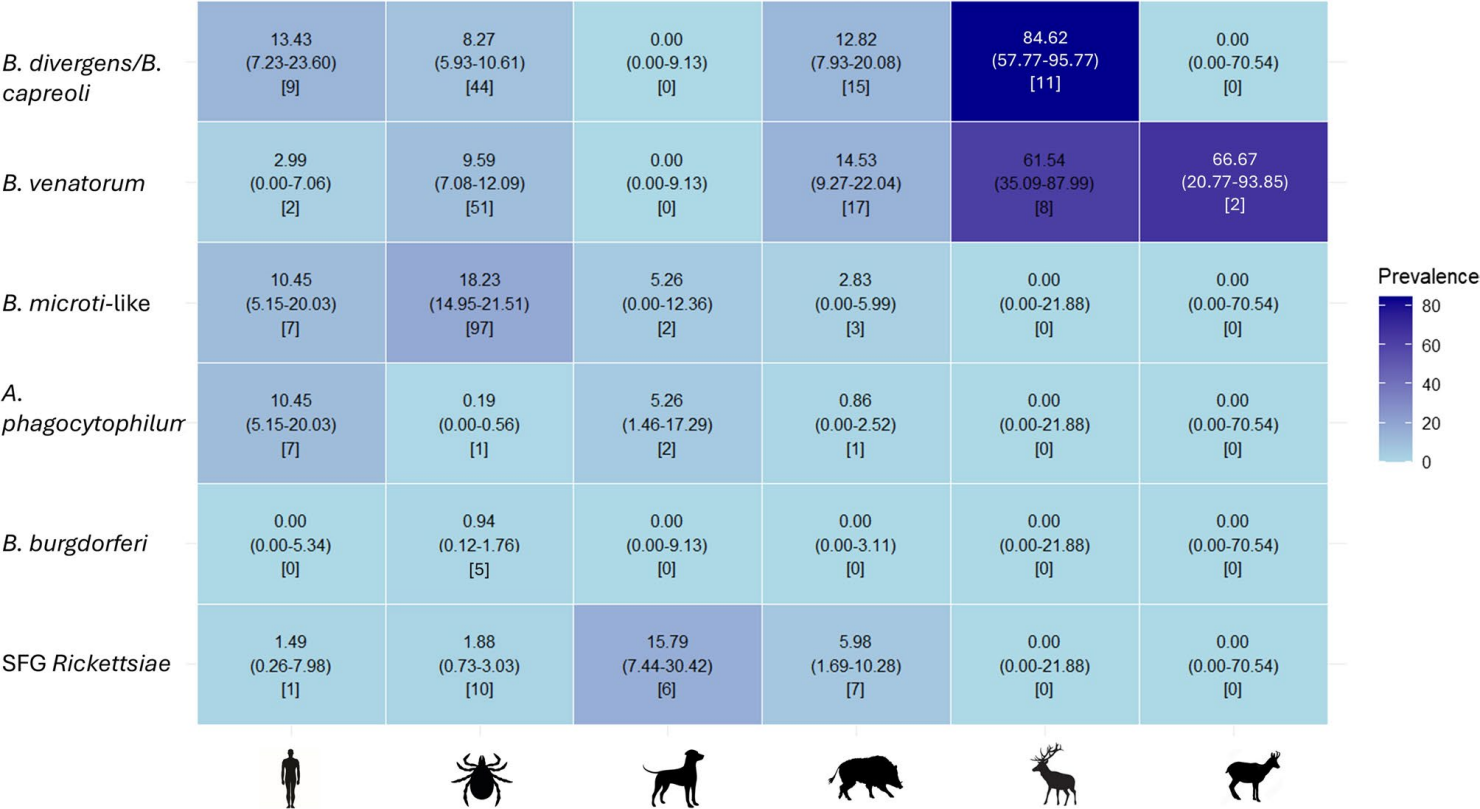
Heatmap of the prevalences of the detected microorganisms across groups. In round brackets, 95% C.I.; in square brackets, the number of positive specimens or ticks. In order: rural inhabitants, questing ticks, dogs, wild boar, deer and chamois

**Fig. 2:**
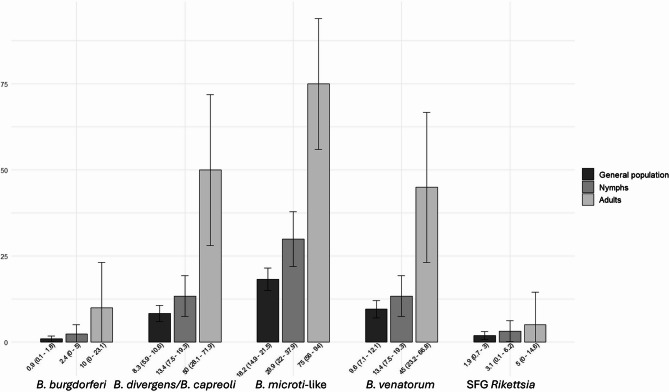
MIR, expressed as a percentage, of the targeted pathogens in the general tick’s population (including all species and developmental stages), nymphs and adults. Below each bar, the MIR value with 95% C.I. We do not report *A. phagocytophilum* as it was only recorded in larvae

*B. microti*-like was most prevalent (18.23%, Fig. 1), followed by *B. venatorum* (9.59%) and *B. divergens/B. capreoli* (8.27%); SFG *Rickettsiae* and *B. burgdorferi* s.l. were less frequent (respectively, 1.88% and 0.94%), and one pool was positive for *A. phagocytophilum*. Patterns in *I. ricinus*, the key zoonotic vector, paralleled overall tick data. Adults showed higher MIR than nymphs and both exceeded prevalence in the general tick’s population analysed (Fig. 2). No adults or nymphs carried *A. phagocytophilum*.

In vertebrate hosts, *B. burgdorferi* s.l. was absent (Fig. 1).

#### Rural inhabitants

*B. divergens/B. capreoli* prevailed (13.43%), followed by *B. microti*-like and *A. phagocytophilum* (both at 10.45%). Sequencing confirmed five *(A) phagocytophilum* cases (93–100% coverage; 95.5–100% identity to KP245908.1; accession numbers PQ657427.1, PQ657428.1, PQ657431.1–PQ657433.1). Two participants were positive for *(B) venatorum*, one for SFG *Rickettsia*. Sequenced SFG *Rickettsia* matched *R. monacensis* (100% coverage; 97–100% identity to LN794217.1; PQ684452). According to statistical analysis, *B. divergens/B. capreoli* prevalence was higher than SFG *Rickettsiae* (*p* = 0.02).

#### Dogs

The highest prevalence was recorded for SFG *Rickettsia* (15.76%, Fig. 1), followed by *B. microti*-like and *(A) phagocytophilum*, detected in two individuals each; *(B) divergens/B. capreoli* and *B. venatorum* were not detected.

#### Wildlife

SFG *Rickettsia*, *(A) phagocytophilum* and *(B) microti*-like occurred only in wild boar. *B. venatorum* prevalence was highest in red deer (61.54%) and chamois (66.67%), followed by wild boar (14.53%); *B. divergens/B. capreoli* peaked in red deer (84.62%), was absent in chamois and lower in wild boar (12.82%). Following statistical analysis, in wild boar *B. microti*-like prevalence was lower than *B. divergens/B. capreoli* (*p* = 0.012) and *B. venatorum* (*p* = 0.002), and *(A) phagocytophilum* was lower than *(B) divergens/B. capreoli* (*p* = 0.002), *B. venatorum* (*p* < 0.001), and SFG *Rickettsiae* (*p* = 0.04).

### Biomolecular detection of microorganisms – between-groups comparison

#### B. divergens/B. capreoli

Red deer had significantly higher prevalence than rural inhabitants, wild boar, and questing ticks (all *p* < 0.001); no other group differences were significant.

#### B. venatorum

Wild ungulates had significantly higher prevalence than rural inhabitants (wild boar *p* = 0.01; red deer *p* < 0.001; chamois *p* = 0.007). Red deer exceeded wild boar (*p* < 0.001), and questing ticks were lower than red deer (*p* < 0.001) and chamois (*p* = 0.027).

#### B. microti-like

Questing ticks had significantly higher prevalence than dogs (*p* = 0.045) and wild boar (*p* < 0.001); rural inhabitants also exceeded wild boar (*p* = 0.047).

#### A. phagocytophilum

Rural inhabitants showed higher prevalence than wild boar (*p* = 0.004) and questing ticks (*p* < 0.001), and dogs exceeded ticks (*p* = 0.013).

#### SFG Rickettsiae

Prevalence was highest in dogs, significantly exceeding rural inhabitants (*p* = 0.009) and ticks (*p* < 0.001); wild boar also had higher prevalence than ticks (*p* = 0.02).

Analysis of the dog owners’ questionnaires revealed no significant association (*p* < 0.05) between PCR positivity and antiparasitic use or the weekly frequency of outdoor activities.

## Discussion

Our findings underscore the circulation of key tick-borne zoonotic pathogens across distinct host groups involved in their transmission. Except for *B. burgdorferi* s.l., which was only detected in questing ticks, rural inhabitants tested positive for all other investigated pathogens. Notably, *B. divergens/B. capreoli* and *B. venatorum* exhibited substantial prevalence in wild ruminants, while *B. microti*-like organisms were sporadically detected in wild boar and domestic dogs. Regarding SFG *Rickettsia*, the highest prevalence was observed in dogs, although occasional detections also occurred in wild boar. *A. phagocytophilum* was found in questing ticks and wildlife at a low prevalence but showed markedly higher detection rates in humans and domestic dogs.

Human babesiosis has been recognized in Europe as an emerging zoonosis [44]. A serosurvey conducted in Northern and Central Italy to investigate the presence in humans of antibodies against *Babesia* species has detected a high reactivity to *B. microti* and *B. divergens* antigens in foresters and hunters, reaching respectively 32% and 12% [45]. Although all participants in our study were healthy individuals without symptoms, we identified a notable prevalence of *B. divergens/B. capreoli*, being *B. divergens* the most common zoonotic *Babesia* species across Europe, and, in addition, we detected lower yet relevant prevalences of *B. microti/microti*-like and *B. venatorum*, confirming that also these zoonotic species are circulating within the human population at notable levels. Zoonotic Babesia species are indeed often present in ticks removed from humans, with MIR reaching up to 31% [5], a finding which is also remarked by our study results, making questing ticks good targets for surveillance of such pathogens.

For *B. divergens/B. capreoli* and *B. venatorum*, red deer and chamois are noted by our study results as frequent hosts. Indeed, *B. divergens* has been found with relevant prevalences, from 5 to 27%, in red deer and cervids [13, 14, 46], and *B. venatorum* has been documented in roe deer [14, 47–49] and chamois [37]. Likewise, *B. venatorum* has been reported in deer species with low to moderate prevalences [48, 50, 51] Given that these species are subject to regulated hunting plans, the relative ease of obtaining spleen samples from them makes them suitable candidates for inclusion in wildlife-based surveillance programs for zoonotic babesia species. However, surveillance in cervid species should target genomic regions of *Babesia* that contain SNPs distinguishing *B. capreoli* from the zoonotic *B. divergens*, as the former, though non-zoonotic, can be detected at high prevalence (up to 46%) in deer species [47, 48]. The role of wild boar in the transmission of zoonotic babesia species, and its relevance to surveillance actions, is still uncertain: while their detection is still uncommon, *B. vulpes* (now classified as *B. microti*-like) has been reported in Italy [52], as well as *B. divergens* in the Czech Republic [53], findings which corroborate the ones of our study but still lead to an inconclusive opinion about their role for the circulation of these pathogens. A relevant exposure of hunting dogs to *B. microti* was remarked in Spain, 15–60% [12, 54], France, 0.7% [55] and Serbia, 10% [56]. Particularly, the presence of clinical cases in dogs [12] remarks the value of surveillance for *B. microti*-like aimed not only at defining the ultimate exposure risk for humans through holistic approaches, but also the risk of developing a disease for pet dogs.

The high prevalence of SFG *Rickettsiae* in dogs detected in this study is consistent with results obtained elsewhere: a study on ticks removed from 221 dogs in different Italian locations has detected SFG *Rickettsiae* as the pathogen present with the highest (51.7%) prevalence [57]. In the present work, this finding may suggest a “domestic” cycle of SFG *Rickettsiae* besides the natural environment, supported by the fact that the primary vector of SFG *Rickettsiae* is ticks of genera *Rhipicephalus* , which is more commonly found in domestic environments. Reports of SFG *Rickettsia* species in wild boar are documented in the literature (e.g. *R. monacensis* by [58], along with detections of relevant prevalences in tick species collected from wild boar [59], findings which align with our results.

Although the detection of circulating *B. burgdorferi* s.l. DNA with an end-point PCR in the blood is sometimes inconclusive due to transient or too low spirochaetaemia [60–62], the identification of the spirochete in ticks highlights the ongoing exposure risk for humans and supports the use of ticks as optimal targets for surveillance efforts. The absence of positive spleen samples from wild ungulates may either corroborate their non-significant role as hosts or again suggest a non-appropriate tissue matrix. However, opportunistic sampling of other tissues such as cerebrospinal fluid or lymph nodes would require an increased effort of specialists to collect samples and would not be feasible through a citizen science approach involving the participation of hunters and hunting associations.

Eight clusters encompassing five ecotypes of *A. phagocytophilum* have been identified so far, according to the literature. Among these, Cluster I is recognized as zoonotic and has been detected in wild ungulates, including deer species and wild boar, raising suspicions of a zoonotic role for these species, along with domestic animals such as dogs and livestock [63, 64]. Despite the potentiality of harbouring zoonotic ecotypes of the pathogen, of all tested culled wildlife only one individual was detected as positive for *A. phagocytophilum*. Considering that there is a certain concordance between environmental ticks and wildlife and a notable difference in humans and dogs, this could again indicate some different sources of infections for people and their pets. Additionally, it should be noted that *A. phagocytophilum* often exhibits fluctuating detectability through biomolecular techniques [65, 66], which may have influenced the low prevalence detected by our PCR techniques. On a final note, studies conducted in nearby areas have also detected a low prevalence of *A. phagocytophilum* in ticks [67, 68].

Human cases of babesiosis, anaplasmosis and rickettsiosis are anecdotal in most European areas, including the one object of this study, and this seems in contrast with the prevalence of circulating DNA we observed in animals, vectors, and healthy volunteers. While asymptomatic infections have been observed, it is possible that these TBZs are largely underdiagnosed (for lack of knowledge and limited capacity for laboratory diagnosis) and underreported. Since the initiation of systematic nationwide data collection in January 2011, the United States has reported an increase in human babesiosis cases (CDC, https://www.cdc.gov/babesiosis/maps-graphs/index.html, accessed on 03/02/2025). Variations in reported cases may not directly reflect true changes in disease incidence but could be influenced by clinician awareness and state-specific public health resources and reporting requirements (CDC, 2022). This underscores the necessity of ongoing surveillance to generate robust epidemiological data and enhance awareness. With regard to wild hosts, this study focused on game ungulates, given their suitability for systematic sampling for the accessibility of their samples. However, the absence of *B. burgdorferi* s.l. and the limited positivity for SFG *Rickettsiae* and *B. microti*-like organisms highlight the importance of including non-game species, such as small mammals, in surveillance programs, as they are recognized reservoirs or key hosts for several tick-borne pathogens [1].

Wildlife plays a key role in the maintenance of certain TBZ, as highlighted by our findings on *Babesia* species, for instance. Wild ungulates, in particular, can contribute not only to the circulation of these pathogens [68] but also to the maintenance of tick populations [17]. Their increasing densities, driven by factors such as land abandonment and rewilding, may elevate the risk of human exposure to TBZ. These dynamics should be carefully considered in the development of wildlife management. 

## Conclusions

The rising popularity of outdoor activities may facilitate the circulation of TBZ pathogens from wildlife to humans, with an expanded and more frequent use of shared spaces and an increase in vector distribution and abundance, demanding appropriate surveillance. Although our results show that targeting questing ticks is often the most suitable method for TBZ surveillance, wildlife hosts can serve as a valuable complement for certain pathogens. For example, wild game species can be particularly informative for monitoring *Babesia* spp., while for others, such as SFG *Rickettsia* and *B. burgdorferi s.l.*, additional sampling of diverse host species is recommended. While the detection of TBZ in less common host species can contribute to the existing literature (such as *B. microti*-like in wild boar or dogs, or SFG *Rickettsia* in wild boar), the role of this hosts is still unclear.

## Supplementary Information

Supplementary Material 1. Supplementary figure. 1. Satellite image of the C.A. CN3 with reference to its position in Italy. Supplementary Table 1. PCR protocols implemented in the present study, with primers and literature sources from where they were taken, and thermic profile and mix adapted to the present study.

## Data Availability

The datasets used and/or analysed during the current study are available from the corresponding author on reasonable request.
