# Supplementary material for: A one-health approach to surveillance of tick-borne pathogens across different host groups
Source: BMC Vet Res. 2025 Oct 2;21:553. doi: 10.1186/s12917-025-04983-7 (PMC12492514; doi:10.1186/s12917-025-04983-7)
Supplement: Supplementary file 1 — Supplementary Material 1. Supplementary figure. 1. Satellite image of the C.A. CN3 with reference to its position in Italy. Supplementary Table 1. PCR protocols implemented in the present study, with primers and literature sources from where they were taken, and thermic profile and mix adapted to the present study. [file 12917_2025_4983_MOESM1_ESM.docx]

**Supplementary material to:** Vada R., Zanet S., Trisciuoglio A., Varzandi A.R., Calcagno A., Ferroglio E. *A One-Health approach to surveillance of tick-borne pathogens across different host groups.*

**Supplementary Figure 1.** Satellite image of the C.A. CN3 with reference to its position in Italy.


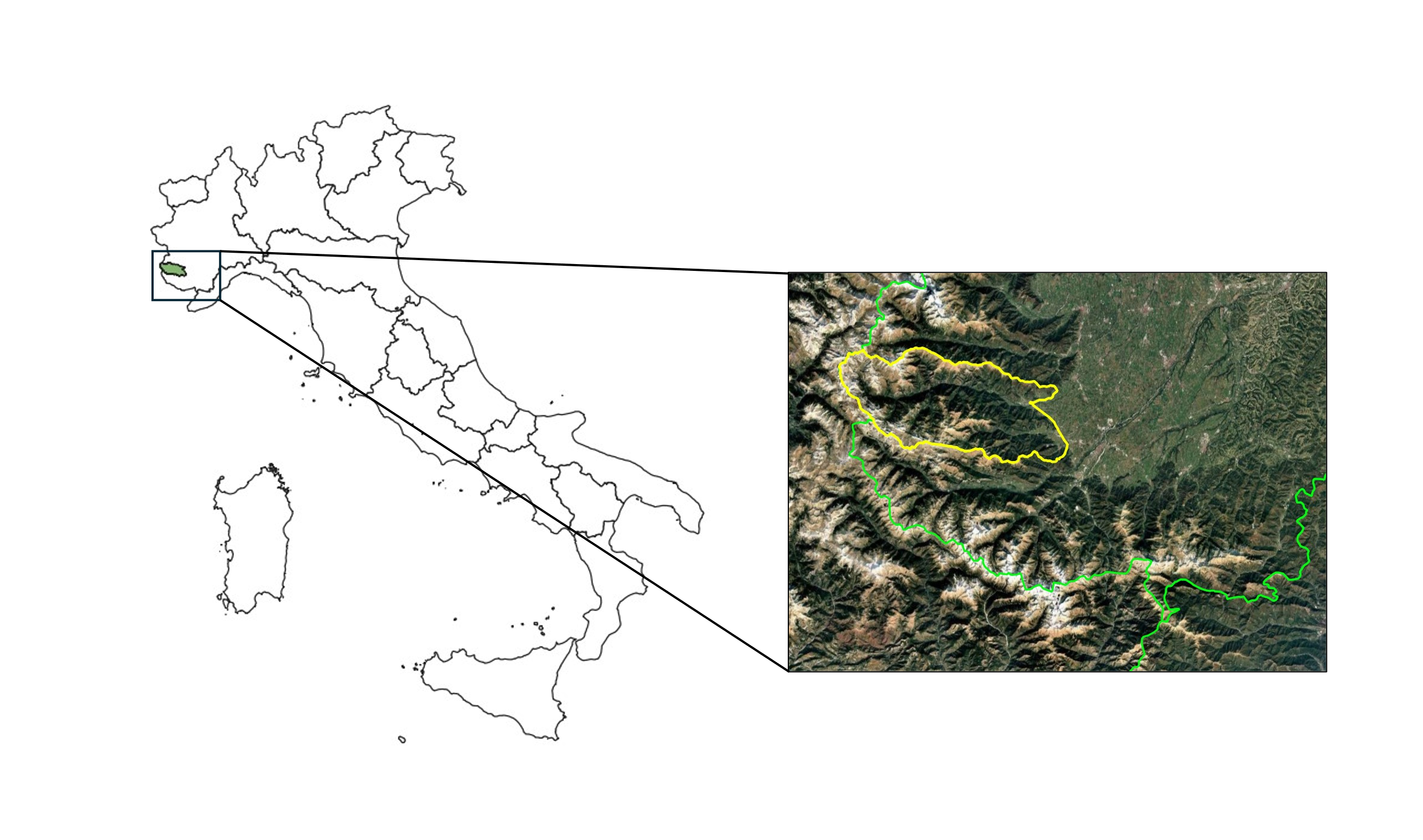


**Supplementary Table 1.** PCR protocols implemented in the present study, with primers and literature sources from where they were taken, and thermic profile and mix adapted to the present study.

| Pathogen | Primers (5’ – 3’) | Thermic profile | Reaction mixture  (25 ul) |
| --- | --- | --- | --- |
| *B. divergens* | *18s* ^[[1]](#footnote-1)^  Forward: GTTTCTGMCCCATCAGCTTGAC  Reverse: CAATATTAACACCACGCAAAAATTC | 95°C - 5’  40 cycles:  94°C - 30”  55°C - 20”  72°C - 30”  72°C - 10’ | PCR Master Mix: 1X (Promega Corporation, WI, USA)  Primers: 10 pmol  DNA: 1 μl |
| *B. microti* | *18s* ^[[2]](#footnote-2)^  Bab1:  CTTAGTATAAGCTTTTATACAGC  Bab4: ATAGGTCAGAAACTTGAATGATACA | 95°C - 5’  40 cycles:  94°C - 30”  51°C - 20”  72°C - 30”  72°C - 10’ | PCR Master Mix: 1X (Promega Corporation, WI, USA)  Primers: 10 pmol  DNA: 1 μl |
| *B. venatorum* | *18s* ^[[3]](#footnote-3)^  Bab_EU_RNA18S_F: GCGCGCTACACTGATGCATT  Bab_EU_RNA18S_R: CAAAAATCAATCCCCGTCACG  Bab_EU_RNA18S_P: CATCGAGTTTAATCCTGTCCCGAAAGG | Preamplification:  95°C - 5’  20 cycles:  94°C - 30”  57°C - 1’  72°C - 10’ | PCR Master Mix: 1X (Promega Corporation, WI, USA)  Primers: 22.5 pmol  DNA: 5 μl |
|  |  | 95°C - 10’  45 cycles:  95°C - 15”  60°C - 1’ | Taqman Universal: 1X (ThermoFisher, MA, USA)  Primers: 22.5 pmol  Probe: 6.25 pmol  Template: 5 μl |
| Anaplasmataceae | *16S rRNA* ^[[4]](#footnote-4)^  PER1: TTTATCGCTATTAGATGAGCCTATG  PER2:  CTCTACACTAGGAATTCCGCTAT | 95°C - 15’  40 cycles:  94°C - 1”  52.4°C - 45”  72°C - 1”  72°C - 10’ | HotStarTaq DNA Polymerase: 2.5 U (Qiagen, Hilden, Germany)  Primers: 25 pmol  MgCl2: 0.5 mM  DNA: 1 μl  dNTPs mix: 0.2 mM (Sigma-Aldrich, St. Louis, MO, USA)  PCR buffer: 10X |
| *A. phagocytophilum* | Gene: *groEL* ^[[5]](#footnote-5)^  EphplgroEL(569)F: ATGGTATGCAGTTTGATCGC  EphplgroEL(1193)R:  TCTACTCTGTCTTTGCGTTC | 40 cycles:  94°C - 30”  55°C - 30”  72°C - 45” | HotStarTaq DNA Polymerase: 2.5 U (Qiagen, Hilden, Germany)  Primers: 25 pmol  DNA: 2.5 μl  dNTPs mix: 0.2 mM (Sigma-Aldrich, St. Louis, MO, USA)  PCR buffer: 10X |
| *B. burgdorferi* s.l. | *spacer region between 5S and 23S rRNA genes* ^[[6]](#footnote-6)^  23SN1:  ACCATAGACTCTTATTACTTTGAC  23SC1:  TAAGCTGACTAATACTAATTACCC  23SN2:  ACCATAGACTCTTATTACTTTGACCA  5SCB:  GAGAGTAGGTTATTGCCAGGG | 95°C - 15’  Touch down:  94°C - 20”  62°C - 30” (-0.5°C every cycle)  72°C - 30”  25 cycles:  94°C - 20”  55°C - 30”  72°C - 30”  72°C - 10’ | HotStarTaq DNA Polymerase: 2.5 U (Qiagen, Hilden, Germany)  Primers: 20 pmol  DNA: 2.5 μl  dNTPs mix: 0.2 mM (Sigma-Aldrich, St. Louis, MO, USA)  PCR buffer: 10X |
| SFG Rickettsia | *surface protein rOmpA* ^[[7]](#footnote-7)^  190-70:  ATGGCGAATATTTCTCCAAAA  90-701:  GTTCCGTTAATGGCAGCATCT | 95°C - 15’  40 cycles:  94°C - 30”  55°C - 30”  72°C - 1’  72°C - 10’ | HotStarTaq DNA Polymerase: 2.5 U (Qiagen, Hilden, Germany)  Primers: 7 pmol  DNA: 5 μl  dNTPs mix: 0.2 mM (Sigma-Aldrich, St. Louis, MO, USA)  PCR buffer: 10X |

1. Heidi Hilpertshauser et al., “Babesia Spp. Identified by PCR in Ticks Collected from Domestic and Wild Ruminants in Southern Switzerland,” *Applied and Environmental Microbiology* 72, no. 10 (2006): 6503–7. [↑](#footnote-ref-1)
2. D H Persing et al., “Detection of Babesia Microti by Polymerase Chain Reaction,” *Journal of Clinical Microbiology* 30, no. 8 (1992): 2097–2103. [↑](#footnote-ref-2)
3. Lorraine Michelet et al., “High-Throughput Screening of Tick-Borne Pathogens in Europe,” *FRONTIERS IN CELLULAR AND INFECTION MICROBIOLOGY* 4 (2014), https://doi.org/10.3389/fcimb.2014.00103. [↑](#footnote-ref-3)
4. Elena Battisti et al., “Molecular Survey on Vector-Borne Pathogens in Alpine Wild Carnivorans,” *Frontiers in Veterinary Science* 7, no. January (2020): 1–9, https://doi.org/10.3389/fvets.2020.00001; Jesse L Goodman et al., “Direct Cultivation of the Causative Agent of Human Granulocytic Ehrlichiosis,” *New England Journal of Medicine* 334, no. 4 (1996): 209–15. [↑](#footnote-ref-4)
5. Alberto Alberti et al., “Anaplasma Phagocytophilum, Sardinia, Italy,” *Emerging Infectious Diseases* 11, no. 8 (2005): 1322. [↑](#footnote-ref-5)
6. S G Rijpkema et al., “Simultaneous Detection and Genotyping of Three Genomic Groups of Borrelia Burgdorferi Sensu Lato in Dutch Ixodes Ricinus Ticks by Characterization of the Amplified Intergenic Spacer Region between 5S and 23S RRNA Genes,” *Journal of Clinical Microbiology* 33, no. 12 (1995): 3091–95. [↑](#footnote-ref-6)
7. Veronique Roux, Pierre-Edouard Fournier, and Didier Raoult, “Differentiation of Spotted Fever Group Rickettsiae by Sequencing and Analysis of Restriction Fragment Length Polymorphism of PCR-Amplified DNA of the Gene Encoding the Protein ROmpA,” *Journal of Clinical Microbiology* 34, no. 9 (1996): 2058–65. [↑](#footnote-ref-7)
